# Distinct Gut Microbiota Signatures in Mice Treated with Commonly Used Food Preservatives

**DOI:** 10.3390/microorganisms9112311

**Published:** 2021-11-07

**Authors:** Ravinder Nagpal, Nagaraju Indugu, Prashant Singh

**Affiliations:** 1 Department of Nutrition & Integrative Physiology, College of Health & Human Sciences, Florida State University, Tallahassee, FL 32306, USA; 2 Center for Animal Health and Productivity, Department of Clinical Studies, School of Veterinary Medicine, University of Pennsylvania, Kennett Square, PA 19348, USA; indugun@vet.upenn.edu

**Keywords:** benzoate, food additives, food preservation, intestine, microbiota, microflora, potassium sorbate, sodium nitrite

## Abstract

Diet is one of the most important factors regulating and influencing the composition of our gut microbiome, but the specific effects of commonly used antimicrobial agents i.e., food preservatives present within foods, are not completely understood. In this study, we examined the effect of the three widely used food-grade preservatives i.e., benzoic acid, potassium sorbate, and sodium nitrite, in recommended levels, on the gut microbiota diversity and composition in a mouse model. The analysis of β-diversity reveals distinct signatures of the gut microbiota between mice consuming different preservatives. Further analyses of α-diversity indices also show that the three preservatives induce specific patterns of microbial diversity, with diversity being lowest in mice consuming potassium sorbate. In terms of bacterial abundance, each of the three preservatives demonstrated unique microbial signatures, mainly affecting the proportions of bacterial taxa belonging to *Bacteroidetes*, *Verrucomicrobia*, and *Proteobacteria*. Specifically, we find the increased proportion of *Bacteroides*, *Blautia*, *Ruminococcus*, *Oscillospira*, and *Dorea* in mice fed with benzoate; increased abundance of *Firmicutes*, *Turicibacter*, and *Alkaliphilus* by sodium nitrate; and increased proportion of *Parabacteroides* and *Adlercreutzia* by potassium sorbate. The findings improve our understanding of how food-grade preservatives may influence the gut microbiota composition and diversity and should facilitate prospective studies investigating diet-microbiome interactions in relation to intestinal and metabolic health.

## 1. Introduction

Our gastrointestinal tract is inhabited by a highly diverse and complex community of microbes (the gut microbiome), which plays a fundamental role in our immune, cardiometabolic and neurocognitive health [1,2,3]. Of various factors influencing our gut microbiome, diet is one of the most prominent elements that strongly regulates and shapes the diversity and composition of the gut microbiome [1,4]. Research over the past two decades has consistently evidenced how abnormal perturbations in the gut microbiome (gut dysbiosis) due to imprudent or unhealthy dietary patterns such as those with high levels of saturated fat, refined carbohydrates, or highly processed foods can lead to increased predisposition to various intestinal disorders and extra-intestinal pathologies [2,5]. While a significant focus of diet-microbiome research has been on dietary macronutrients (e.g., fiber, fatty acids, protein sources) and micronutrients (e.g., vitamins, iron, magnesium, calcium, zinc, etc.), the role and effect of food preservatives, which have become an increasingly prevalent and regular component of our modern-day dietary lifestyle with increased intake of processed foods, on gut microbiome remain relatively underexplored.

The United States Food and Drug Administration (FDA) defines antimicrobial agents as “substances used to preserve food by preventing the growth of microorganisms and subsequent spoilage, including fungistats, and mold and rope inhibitors” [6]. Food preservatives are naturally occurring or synthetically produced substances that are added (alone or in combination with other preservation processes) to prevent microbial growth and increase the shelf life of food products. The commonly used food preservatives are benzoic acid, sorbic acid, ascorbic acid, sodium nitrite, calcium propionate, sulfites, and disodium ethylenediaminetetraacetate (EDTA) [7,8]. The federal regulatory agencies regulate the limits of these preservatives in specific food products. The addition of these additives must be labeled on the product. Although the type and concentration of these preservatives are generally regarded as safe for human consumption mainly in terms of toxicity and mutagenicity, some studies have hinted that increased consumption of preservatives due to increasing processed food consumption may be detrimental to human gut health. For example, studies have shown that food emulsifiers, which are commonly used ingredients in processed foods, can promote gut leakiness [9], thereby increasing the predisposition to inflammatory bowel disease [10], colitis [11], adiposity [11], and metabolic syndrome [11]. Given that all these disorders are closely associated with gut microbiome dysbiosis, it becomes pertinent to determine the impact of exposure to these food preservatives in regulated doses on the gut microbiota composition [12]. Moreover, a significant amount of food preservatives is unabsorbed in the human gastrointestinal tract, and hence such preservatives are likely to directly interact with the gut microbes.

Some studies have separately reported the effect of preservatives and emulsifiers on gut microbiota composition [11,12,13,14,15,16], but the individual effects of some of the common preservatives on the intestinal microbiome in a single setting remain unstudied. Therefore, the aim of this study was to demonstrate the effect of three most readily used food preservatives, i.e., benzoic acid (BA), sodium nitrite (SN), and potassium sorbate (PS), in doses equivalent to that recommended safe in humans, on the gut microbiota diversity and composition in mice.

## 2. Materials and Methods

### 2.1. Animals

All experiments and procedures were performed in accordance with the guidelines of the Institutional Animal Care and Use Committee (IACUC; protocol #1807). Thirty-two C57BL/6 J mice (stock #000664; age 5-weeks; male) were purchased from the Jackson Laboratory (Bar Harbor, ME, USA). Animals were allowed to acclimatize for one week, during which the animals were maintained on standard laboratory rodent diet 5001 *ad libitum*.

### 2.2. Intervention

Following the acclimatization period, the mice were randomly divided into four experimental groups (*n* = 8 per group). Three experimental groups received one of the following treatments: (i) 0.3% potassium sorbate (3000 PPM), (ii) 0.1% benzoic acid (1000 PPM), or (iii) 0.05% sodium nitrate (500 PPM). The fourth group acted as a control (CTL) group. Each preservative was individually added to the laboratory rodent diet 5001 (LabDiet). The diets were formulated in collaboration with and procured from the TestDiet Inc. (St. Louis, MO, USA) as follows: (i) LabDiet#5001 with no preservative; (ii) Modified-LabDiet#5001 with 0.3% potassium sorbate (5WKM); (iii) Modified-LabDiet#5001 with 0.1% benzoic acid (5WKL); and (iv) Modified-LabDiet#5001 with 0.05% sodium nitrate (5WKN). We monitored the food intake throughout the study and found no significant difference in food intake between different groups. On average, the food intake was 4.63 (CTL), 4.15 (BA), 3.45 (PS), and 3.15 (SN) g/mouse/day, which corresponded to the intake of 0.019, 0.049, and 0.007 mg preservative per gm body weight per day for BA, PS, and SN, respectively. Animals were fed *ad libitum* for 12 weeks, with free access to drinking water. Bodyweight and diet-intake were measured periodically throughout the intervention.

### 2.3. Microbiome Measurement

The gut microbiota was examined as per our previously described methods [17,18,19]. Briefly, the Earth Microbiome Project (EMP) benchmarked protocol (http://www.earthmicrobiome.org; date accessed: 30 March 2021) was adopted employing a barcoded high-throughput sequencing approach as described by Caporaso et al. [20]. Briefly, the fecal samples from individual mice were stored at −80 °C as soon as possible after collection until microbial DNA extraction. Bacterial genomic DNA from 200 mg feces was extracted using a PowerFecal DNA kit (Qiagen, Valencia, CA, USA) according to the manufacturer’s instructions. To avoid the influence of DNA extraction and the PCR conditions and primers on community composition recovered by amplicon sequencing, all samples were processed simultaneously and identically to minimize biasing the microbial community composition. The V4 hypervariable region of the 16S rDNA gene was PCR-amplified using the universal primers 515F (barcoded) and 806R; the resulting amplicons were purified with AMPure^®^ magnetic purification beads (Agencourt); the purified products were quantified using the Qubit-3 fluorimeter (Invitrogen, Waltham, MA, USA); and the amplicon library was generated according to methods described elsewhere [20]. The purified PCR product was pooled in equimolar concentrations and sequenced on an Illumina MiSeq platform using a 2 × 150 bp reagent kit (Miseq reagent nanokit; Illumina Inc., San Diego, CA, USA) for paired-end sequencing. The sequencing quality control was executed with on-board Miseq Control Software and Miseq Reporter (Illumina Inc., San Diego, CA, USA). The resultant paired-end reads were de-multiplexed and were assigned to individual samples based on their unique barcode. The obtained sequences generated were de-multiplexed, quality-filtered, clustered, and analyzed using QIIME software package (ver. 1.9.1) [21] according to our previously described workflow [17,18,22]. Operational taxonomic units (OTUs) were chosen by open reference OTU picking based on 97% sequence similarity to the Greengenes database [21]. To avoid bias of different sequencing depth, the OTU tables were rarefied to the lowest number of sequences per sample for computing diversity metrics. α diversity measures including observed OTUs, Shannon index, and Simpson index were computed within QIIME. β diversity of the microbiome was analyzed using principal coordinate analysis (PCoA) of the unweighted and weighted Unifrac distance (using EMPeror version 0.9.3-dev). Bacterial taxonomy assignment was calculated within QIIME using default settings to compare the bacterial diversity and abundance between the different groups. To avoid the bias of sequencing errors or low-level contaminations, the OTUs with very small read count (less than 4) in very few samples (less than 10% prevalence) were filtered out from the subsequent analyses. The data of taxon abundance were further subjected to the total sum scaling, and the taxa with less than 1% mean relative abundance were excluded from the subsequent downstream analyses. The bacterial community composition of each sample was measured at taxonomic levels of phyla, classes, orders, families, and genera.

### 2.4. Gene Expression Analysis

Animals were euthanized by CO_2_ asphyxiation. Ileum, duodenum, and cecum samples were collected immediately post-euthanasia and kept on dry ice before storage at −80 °C for long-term preservation. mRNA from ileum samples was isolated using Direct-zol™ RNA MiniPrep Kits (Zymo Research, Irvine, CA, USA) following the manufacturer’s instructions. Isolated mRNA samples were reverse transcribed into cDNA using a High-Capacity cDNA Reverse Transcription Kit (Applied Biosystems Foster City, CA, USA). cDNA samples were diluted to 50 ng/µL working concentration and used for gene expression assays. Gene expression of Zo-1, Zo-2, Occludin, and Claudin-2 genes were measured using previously published primers [23,24,25,26].

### 2.5. Statistical Analysis

α-diversity indices and bacterial abundance between different groups were compared using one-way analysis of variance (ANOVA) followed by Dunn’s post hoc analysis and Bonferroni *p*-value corrections. LEfSE (linear discriminatory analysis [LDA] effect size) was used to identify bacterial taxa that drive differences between different groups of mice [27]. Differences in beta diversity were tested by permutational multivariate analysis of variance (PERMANOVA), a permutation-based multivariate analysis of variance to a matrix of pairwise distance to partition the inter-group and intra-group distance. Hierarchical clustering heatmaps depicting the patterns of abundance were constructed within ‘R’ statistical software package (version 3.6.0; https://www.r-project.org/; date accessed: 30 March 2021) using the ‘heatmap.2’ and “ggplots” packages. Random forest supervised learning model was applied on normalized data within R (model_randomForest; 70% training and 30% testing modules; trees = 500) to identify the major taxa whose abundance is affected by different preservatives. Unless otherwise stated, all the values presented herein are means ± SEM. *p* < 0.05 was considered statistically significant unless specified.

## 3. Results

### 3.1. Different Food Preservatives Distinctly Impact the Gut Microbiome Diversity

**Figure 1:**
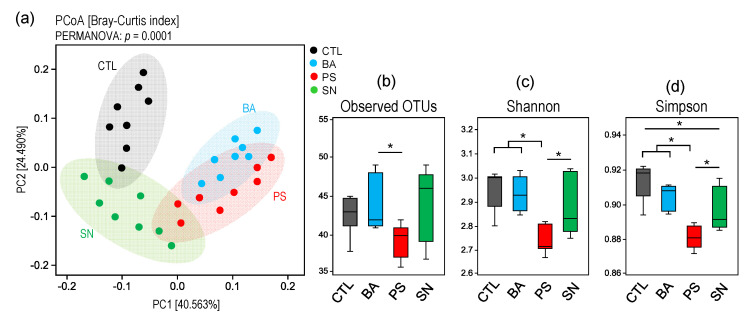
Distinct gut microbiome arrays in mice consuming different preservatives. (**a**) β-diversity (principal coordinate analysis; PCoA) and (**b**–**d**) α-diversity indices in mice consuming benzoate (BA), potassium sorbate (PS), or sodium nitrite (SN) versus control (CTL) mice. * *p* < 0.05.

The analysis of β-diversity (a measure of microbial diversity differences among the groups) of the gut microbiome reveals that the three preservatives induce distinct signatures of the gut microbiome as compared to the control group (Figure 1a). The β-diversity of microbiome signatures of three preservative groups are clustered distinctly from each other as well as from the control counterparts. The microbiomes of BA and PS groups are clustered relatively close to each other, while the SN group is relatively closer to CTL group but still clearly distinct from other groups (Figure 1a). Further analyses of α-diversity (a measure of microbial diversity within samples) indices (i.e., number of OTUs [operational taxonomic units] detected, Shannon index, and Simpson index) also show that the three preservative groups of mice harbor distinct populations of gut microbes indicated by differences in the α-diversity indices of the gut microbiome. The PS group shows the lowest and most distinct pattern in terms of all α-diversity indices (Figure 1b–d). Overall, the α-diversity in BA and SN groups remains similar to that in CTL mice, while the SN mice show a slightly reduced Simpson index as compared to CTL counterparts (Figure 1b–d). In contrast, PS group demonstrates remarkably reduced diversity as compared to all of the other three groups of mice (Figure 1b–d).

### 3.2. Food Preservatives Generate Distinct Microbiota Composition in the Mouse Gut

**Figure 2:**
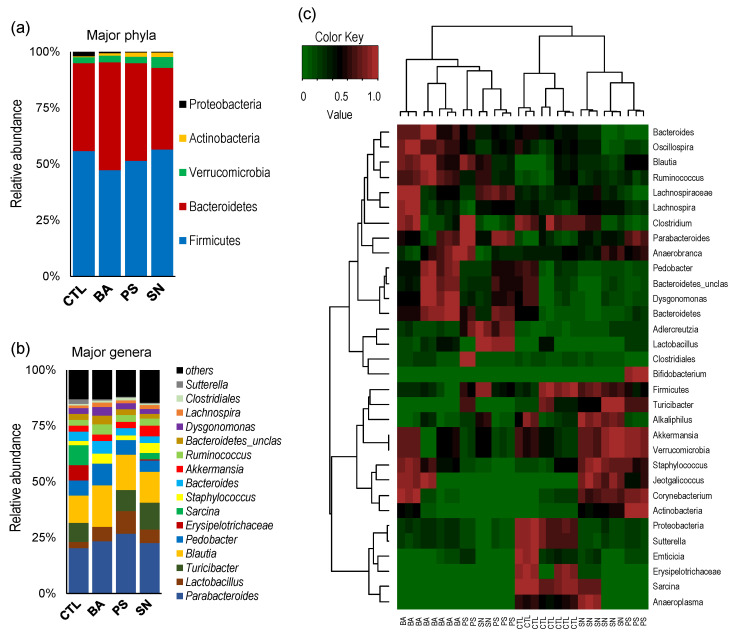
Distinct gut microbiota composition in mice fed with different preservatives. The microbiota composition at the level of major phyla (**a**) and genera (**b**), and the hierarchical clustering heatmap (**c**) depicting distinct spectrums of gut microbiota in mice consuming benzoate (BA), potassium sorbate (PS), or sodium nitrite (SN) versus control (CTL) mice.

The relative abundance of major phyla is found to be slightly distinct in three preservative groups as compared to CTL counterparts as well as to each other (Figure 2a), suggesting that each of these three preservatives induced a unique microbial phyla signature. The BA and PS groups have a slightly increased proportion of phylum *Bacteroidetes*, whereas the SN group has an increased proportion of phylum *Verrucomicrobia* as compared to the CTL group (Figure 2a). The abundance of phylum *Actinobacteria* is slightly higher in all three experimental groups versus the CTL group. Subsequent analyses of relative abundance at the level of bacterial genera also reveal specifically distinct and unique arrays in all the three preservatives groups wherein the differences in the experimental groups are determined largely by the members of the genera *Lactobacillus*, *Blautia*, *Turicibacter*, *Erysipelotrichae*, and *Sarcina* (Figure 2b). The proportion of genera *Lactobacillus* and *Blautia* is increased while that of *Erysipelotrichae* and *Sarcina* is decreased in the three preservative groups versus the CTL group. The abundance of *Turicibacter* is reduced while that of *Bacteroides* and *Ruminococcus* is increased only in the BA group. PS group has the highest increase in the abundance of *Parabacteroides* and *Lactobacillus*, whereas the SN group is characterized by the highest increase in *Turicibacter* and *Akkermansia* versus all the other groups (Figure 2b). In line with these distinct patterns, the combined hierarchal clustering analysis of major bacterial phyla, families, and genera also reveal distinct arrays of clustering among the four groups (Figure 2c), wherein the BA group is clustered far distinctly versus the CTL group while the SN and PS groups have specific overlaps and are clustered close relatively closer to the CTL group. Nevertheless, all the mice in the CTL group are clustered together and distinctly from the three experimental groups, indicating the distinct gut microbiota signatures in these groups.

### 3.3. Mice Treated with Different Preservatives Present Unique Gut Microbiota Signatures

**Figure 3:**
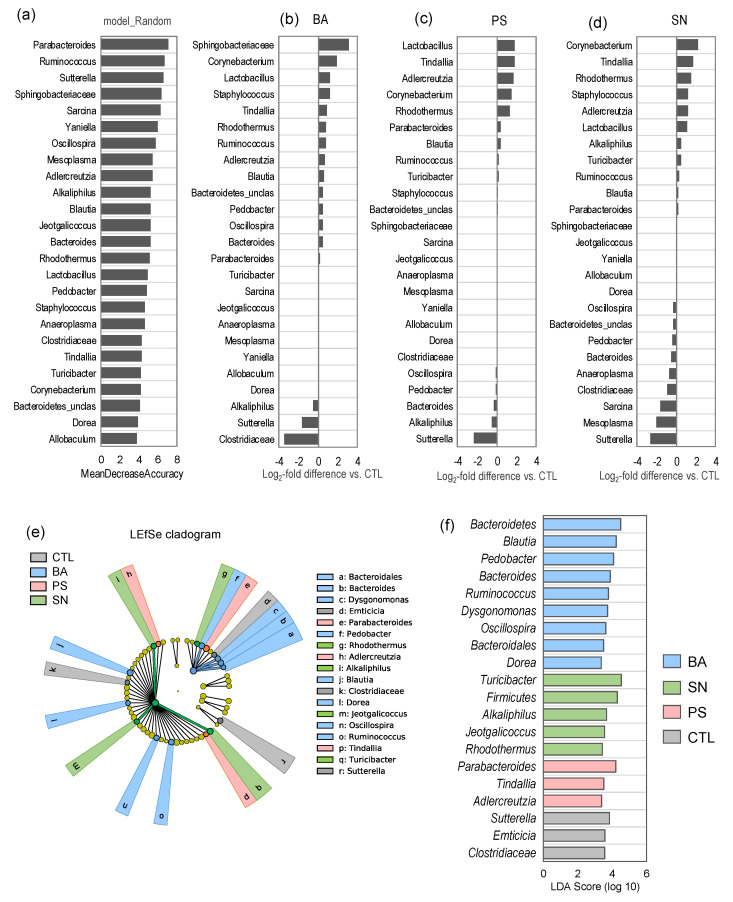
Specific microbiota signatures in mice consuming different preservatives. (**a**–**d**) The random forest graph shows the most predictive bacterial phyla, orders, families, and genera influenced by the intake of specific preservatives. (**e**,**f**) Linear discriminatory analysis (LDA) effect size (LEfSe) cladogram (**e**) and LDA bar plot (**f**) representing the bacterial taxa significantly unique in mice consuming benzoate (BA), potassium sorbate (PS), or sodium nitrite (SN) versus control (CTL) mice.

We then perform the microbiome biomarker discovery algorithm of LEfSe (Linear discriminatory analysis effect size) analysis to distinguish unique bacterial taxa in each preservative group (Figure 3a,b). As shown in the LEfSe-generated cladogram (Figure 3a), all four groups of mice demonstrate clearly distinct microbiota community signatures wherein the magnitude of uniqueness and effect is relatively higher for BA followed by SN and PS. As further simplified by the LDA (linear discriminatory analysis) score graph (Figure 3f), the mice in the BA group harbor a significantly higher proportion of *Bacteroidetes*, *Clostridia*, *Blautia*, *Pedobacter*, *Bacteroides*, *Ruminococcus*, *Dysgonomonas*, *Oscillospira*, and *Dorea* as compared to all of the other three groups. In contrast, the mice treated with SN harbor a high proportion of *Firmicutes*, *Turicibacter*, *Alkaliphilus*, *Jeotgalicoccus*, and *Rhodothermus*, whereas the PS mice are distinguished by a higher abundance of *Parabacteroides, Tindallia* and *Adlercreutzia*. Further, all the three preservative groups have a lower population of *Clostridiaceae*, *Sutterella*, and *Emticicia* as compared to the CTL mice (Figure 3f).

## 4. Discussion

Food preservatives are commonly added to increase the shelf-life of food products. Benzoic acid, potassium sorbate, and sodium nitrate are among the food industry’s top food preservatives. The benzoic acid is added to control the growth of yeast and mold [8]. Their antimicrobial efficacy is associated with microbial membrane disruption, inhibition of metabolic reactions, and accumulation of toxic anions in the microbial cells [28]. Sorbic acid is incorporated in foods to inhibit the growth of yeast, molds, and Gram-negative (e.g., *Campylobacter*, *Clostridium*, *Escherichia coli* O157:H7, *Salmonella*) and Gram-positive foodborne pathogens (e.g., *Staphylococcus*) [8]. Whereas sodium nitrate is highly effective in controlling *Clostridium botulinum* and other *Bacillus* species [8]. The exact mode of action of nitrite against *C. botulinum* is not well characterized [29]; however, nitrite is considered as a precursor to peroxynitrite (ONOO–), which is a strong oxidizing agent [30]. The U.S. Food and Drug Administration permits these preservatives at (0.1%) benzoic acid, (0.1 to 0.3%) potassium sorbate, and (<500 ppm) sodium nitrate concentration [31]. However, the effect of regular ingestion of these preservatives within these concentrations remains underexplored. In this context, this study aimed to evaluate the in vivo influence of these three most commonly used food preservatives on the gut microbiota composition in conventional mice.

In this study, the PS group showed the lowest and most distinct makeup at all three α-diversity level indices (i.e., number of OTUs detected, Shannon index, and Simpson index) (Figure 1b–d). In addition, the Simpson index of α-diversity demonstrates a decline in the bacterial diversity (Figure 1b), with diversity being modestly reduced in the BA group followed by considerably reduced in the SN group while being remarkably reduced in the PS group mice, indicating that the three preservatives induce distinct magnitude of impact on the gut microbiota diversity and composition. A similar reduction in the α diversity measured in terms of Faith’s phylogenetic index has previously been reported in wild-type mice administered with a combination of sodium benzoate (4.8 mg/kg bw/day), sodium nitrite (0.36 mg/kg bw/day), and potassium sorbate (19.0 mg/kg bw/day). However, this study provided these preservatives via drinking water [12].

The gut microbiome β-diversity analysis clearly revealed distinct clusters (Figure 1a) specific for the three preservative groups of mice compared to each other and the control group, indicating different microbiome signatures between mice treated with different preservatives. The treatment and control groups clustered distinctly from each other, with the BA and PS groups samples clustered relatively close to each other. Hrncirova et al. (2019) have reported similar susceptibility of gut microbes at the β-diversity level among wild-type mice administered with a combination of food preservatives in the drinking water. In addition, studies have also reported potent antimicrobial effects of sodium nitrite and its combinations with other common food preservatives (i.e., benzoate, sorbate) on pure culture aerobic and anaerobic bacterial strains of human origin [14]. In our study, the β-diversity analysis showed that the SN group was relatively closer to the CTL group, indicating gut microbiota’s resilience or tolerance towards sodium nitrate. Sodium nitrate is commonly used in processed meat products (i.e., beacon, jerky, lunch meat). It helps meat processors achieve the desired color of red meat products and has a proven track record of being effective against *Clostridium botulinum* [8]. In this study, the SN-treated group showed the highest increase in *Turicibacter* and *Akkermansia*.

Further, the proportion of genera *Lactobacillus* and *Blautia* increased in all three treatment groups, and all the three preservative groups had a lower population of *Proteobacteria*, which is comprised of several Gram-negative and opportunistic pathogens. The data from this in vivo study might indicate that the consumption of potassium sorbate, sodium nitrate, and benzoic acid does not negatively affect the gut commensal/beneficial bacterial genera but reduces the proportion of *Proteobacteria* members. We further observe differences in the abundance of several other bacterial clades typically associated, as commensals, with the murine microbiota, such as *Parabacteroides*, *Lactobacillus*, *Blautia*, *Sarcina*, *Staphylococci*, *Bacteroides*, and *Ruminococci* (Figure 2). These differences are observed even at the highest level of taxonomical classification, i.e., at the phylum level, a reduction in *Firmicutes* and expansion in the abundance of *Bacteroidetes*, *Verrucomicrobia*, or *Actinobacteria* (Figure 2a), thereby indicating specific microbiota spectrums different from that in control mice.

Alterations in microbiota composition have been linked to an altered intestinal epithelial permeability, which may increase or alter the interface of gut bacteria with the host intestinal immune and enteric nervous system. Therefore, to estimate if the effects of food preservatives are seen beyond the microbiota composition, we also measured the gene expression arrays of the intestinal tight-junction markers (Zo-1, Zo-2, Occl, and Clau-2) in the ileal tissues (Supplementary Figure S1) and observed significant or insignificant reduction in the expression of these markers in all the three preservative groups, although the degree of this effect varied according to the class of preservative. However, these effects require further experiments to verify the effect, if any, of these preservatives on the gut microbial-epithelial interplay, which was out of the scope of the present study focused primarily on the microbiota composition. As such, no significant difference was observed in the body weight or diet intake between these groups of mice (Supplementary Figure S1).

The study has a few limitations. One limitation is that the previous studies in this aspect have focused either on the effects of food ingredients on gut microbiota [11,13,16] or evaluated the effects of preservatives on pure culture bacterial strains [14,15], both of which limited our options for data comparisons and discussions therefrom. Another limitation is that we only used male mice for this preliminary study to comply with the three R’s of the guiding principles for the ethical use of animals. It will be interesting to see in prospective studies if these effects on the microbiome vary between male versus female subjects and whether and how these microbiota effects affect any functional feature of the mucosal-associated microbiota and the epithelial membrane integrity and function. Additionally, we used a dose corresponding to that used in food products, but some of these preservatives are produced naturally in plants and might also occur naturally in the human body. It will be an interesting topic for future studies to examine whether and how such inherent preservatives interact with the microbiome in relation to food-related preservatives. Nevertheless, to our knowledge, this is the first study examining the effects of regular intake of these three commonly used preservatives on gut microbiota composition under the same experimental setting. Overall, the data conclude that the intake of potassium sorbate, benzoic acid, and sodium nitrate at recommended levels may induce distinct microbiome signatures specific to the three preservatives, with all three preservatives showing a reduction in *Proteobacteria*, without any major gut dysbiosis. The study will facilitate prospective studies investigating the dietary components and additives in relation to gut microbiota and intestinal health.

## Data Availability

All the raw sequencing data sets have been submitted to the NCBI Sequence Read Archive database under SRA accession number: SUB10382603 and bio-project number PRJNA763607).

## Supplementary Materials

The following are available online at https://www.mdpi.com/article/10.3390/microorganisms9112311/s1, Figure S1: Bodyweight and the ileal mRNA expression profiles of gut permeability markers in mice fed different preservatives.
