# Supplementary material for: Distinct Gut Microbiota Signatures in Mice Treated with Commonly Used Food Preservatives
Source: Microorganisms. 2021 Nov 7;9(11):2311. doi: 10.3390/microorganisms9112311 (PMC8617889; doi:10.3390/microorganisms9112311)

Supplementary Fig. S1. Bodyweight and the ileal mRNA expression profiles of gut permeability markers in mice fed different preservatives. (a) Changes in the body weight of mice consuming benzoate (BA), potassium sorbate (PS), or sodium nitrite (SN) versus control (CTL) mice at the baseline and endpoint of 12-week intervention period. (b) Differences in the mRNA expression levels of gene markers of gut permeability in the ileal tissues of mice after 12-week consumption of benzoate (BA), potassium sorbate (PS), or sodium nitrite (SN) versus control (CTL) mice. *$P<0.05$ vs. CTL; #$P<0.1$ vs. CTL.

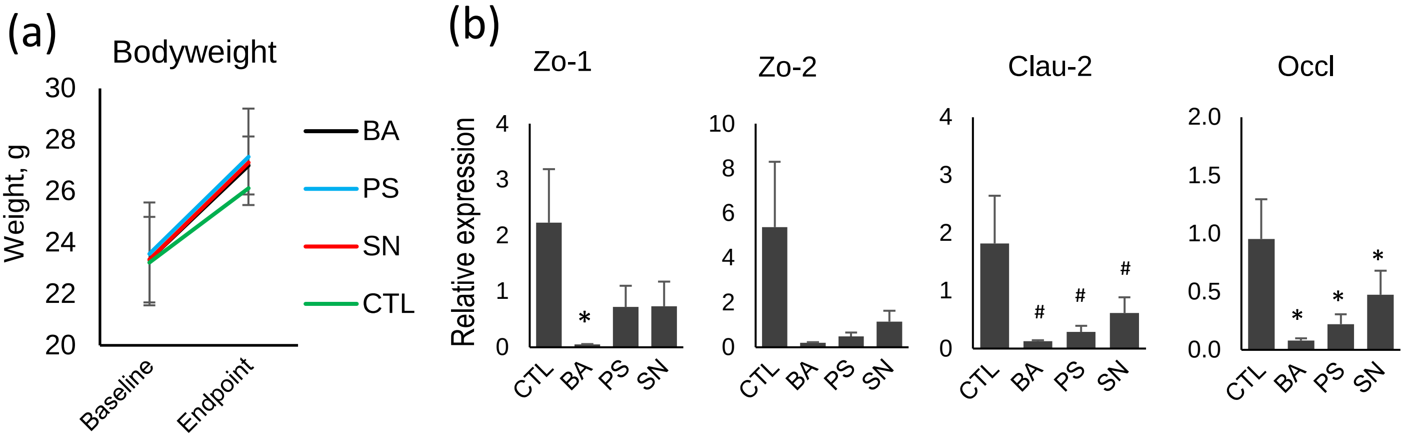

Supplement: Supplementary file 1 [file microorganisms-09-02311-s001.zip › microorganisms-1404522-supplementary.pdf]
